# The development and application of an oncology Therapy-Related Symptom Checklist for Adults (TRSC) and Children (TRSC-C) and e-health applications

**DOI:** 10.1186/1475-925X-14-S2-S1

**Published:** 2015-08-13

**Authors:** Arthur R Williams, David D Williams, Phoebe D Williams, Farrokh Alemi, Hosai Hesham, Blaine Donley, Raya E Kheirbek

**Affiliations:** 1 Center of Innovation on Disability and Rehabilitation Research (CINDRR), James A. Haley Veterans Administration Medical Center, Tampa, FL, 33637 USA; 2 Health Administration and Policy, College of Health and Human Services, George Mason University, Fairfax, VA, 22030 USA; 3 Health Services & Outcomes Research, Children's Mercy Hospitals and Clinics, Kansas City, MO, 64108 USA; 4 School of Nursing, Kansas University Medical Center, Kansas City, KS, 66160 USA; 5 Chief of Performance Improvement, District of Columbia Veterans Administration Medical Center, Washington D.C., 20422 USA; 6 Otolaryngology Section Chief, District of Columbia Veterans Administration Medical Center, Washington D.C., 20422 USA; 7 Quaso LLC, Montross, VA, 22520 USA; 8 District of Columbia Veterans Administration Medical Center, Washington D.C., 20422 USA

## Abstract

**Background:**

Studies found that treatment symptoms of concern to oncology/hematology patients were greatly under-identified in medical records. On average, 11.0 symptoms were reported of concern to patients compared to 1.5 symptoms identified in their medical records. A solution to this problem is use of an electronic symptom checklist that can be easily accessed by patients prior to clinical consultations. *Purpose: *Describe the oncology Therapy-Related Symptom Checklists for Adults (TRSC) and Children (TRSC-C), which are validated bases for e-Health symptom documentation and management. The TRSC has 25 items/symptoms; the TRSC-C has 30 items/symptoms. These items capture up to 80% of the variance of patient symptoms. Measurement properties and applications with outpatients are presented. E-Health applications are indicated.

**Methods:**

The TRSC was developed for adults (N = 282) then modified for children (N = 385). Statistical analyses have been done using correlational, epidemiologic, and qualitative methods. Extensive validation of measurement properties has been reported.

**Results:**

Research has found high levels of patient/clinician satisfaction, no increase in clinic costs, and strong correlations of TRSC/TRSC-C with medical outcomes. A recently published sequential cohort trial with adult outpatients at a Mayo Clinic community cancer center found TRSC use produced a 7.2% higher patient quality of life, 116% more symptoms identified/managed, and higher functional status.

**Discussion, implications, and follow-up:**

An electronic system has been built to collect TRSC symptoms, reassure patients, and enhance patient-clinician communications. This report discusses system design and efforts made to provide an electronic system comfortable to patients. Methods used by clinicians to promote comfort and patient engagement were examined and incorporated into system design. These methods included (a) conversational data collection as opposed to survey style or standardized questionnaires, (b) short response phrases indicating understanding of the reported symptom, (c) use of open-ended questions to reduce long lists of symptoms, (d) directed questions that ask for confirmation of expected symptoms, (e) review of symptoms at designated stages, and (d) alerting patients when the computer has informed clinicians about patient-reported symptoms.

**Conclusions:**

An e-Health symptom checklist (TRSC/TRSC-C) can facilitate identification, monitoring, and management of symptoms; enhance patient-clinician communications; and contribute to improved patient outcomes.

## Background and purposes

In the USA, the incidence of cancer has been increasing for many years, treatment costs rising, and, consequently, aggregate expenditures growing. In recent years, incidence rates of some adult cancers have slowed, but treatment costs continue to rise along with the use of newer and more expensive interventions. Unfortunately, the use of newer interventions and increased survival has brought with them the increased likelihood of adverse effects of treatment affecting patient symptoms and outcomes. Many patients leave treatment due to adverse effects.

Despite the apparent slowing incidence of adult cancers, recent statistics suggest that incidence rates of cancers in children and adolescents are increasing as is treatment costs and concerns about better management of patient treatment symptoms. Recent data indicate that the costs of treating childhood cancers may exceed treatment costs for adults.

### Recognition of the need for improved management of patient symptoms

Symptoms arising from use of oncology therapies require careful monitoring for problems of adjustment to treatment regimens and for identification of adverse effects on patients. Since the 1980s, clinical guidelines in the USA have strongly urged the monitoring of subjectively reported treatment symptoms as stated by patients; however, certain factors have worked against such systematic monitoring. First, the average time spent with patients by physicians during consults is around 19 minutes but frequently less than 15 minutes, which greatly narrows time for conversations. Very limited time may be spent on topics specific to these visits [1]. Second, the clinical interview is often unstructured with patient's being asked "what problems have you had" often without any prompts related to "problems" that may be of special concern to treatment of the patient. Third, at least until recently, the collaborative role of the patient has been lightly regarded in clinical training, in the literature, and in practice. Fourth, although changing under computerization, medical records are often poorly and inconsistently maintained. For these reasons and consistent with anecdotal reports, many observers of health care in the USA believe that patient symptoms associated with therapies were under-identified in medical records. Consequently, a valuable resource for improved treatments and outcomes - symptoms of concern to patients - was being underutilized or even ignored.

One of the earlier studies of the collection and use of patient reported symptoms was an oncology nursing study by Youngblood et al. in 1994 [2]. The study examined the medical records of 91 patients who after clinical consultation were asked to respond to the presence and intensity of any of 37 symptoms that were of concern to them. On average, patients' medical records identified only 1.5 symptoms (range 0-9; SD = 1.6). However, on average, these same patients self-reported 11.0 symptoms of concern to them (range 0-37; SD = 8.0). Many of the unidentified symptoms could have led to substantial changes in therapy and treatment outcomes.

## Methods

### Creation of the Therapy-Related Symptom Checklist for Adults (TRSC)

A year following the above report another study was undertaken to develop a tool or checklist that could be readily used in oncology outpatient clinics. Essential requirements of such a tool are that it be quickly answered, easily understood by patients, not add to burdens faced by worried cancer patients, and be comprehensive in terms of checklisted symptom concerns of patients. If a tool meets all these criteria, it is deemed "clinic friendly" in that it can be readily answered by patients in busy clinics prior to their consultation with physicians or nurses.

The tool used by Youngblood et al. consisted of 37 items or symptoms drawn from Eastern Cooperative Oncology Group (ECOG) documents and the clinical experiences of the authors [3]. It was decided to obtain a large sample using this tool, subject data collected to analysis, and determine whether a clinic friendly checklist could be produced. Two hundred eighty-two patients 18-83 years of age undergoing chemotherapy, radiation, or combined therapies at a cancer center in the Midwest USA answered the 37 item checklist that included spaces for patients to add symptoms if they desired. Few symptoms were added; therefore, these were not included in the analysis. (See Appendix A, additional file 1).

An anti-image correlation matrix was obtained, and measures of sampling adequacy (MSA) and the Kaiser-Meyer-Olkin (KMO) were calculated [4]. Nine of the 37 items (symptoms) had MSA <0.70 and were dropped. The elimination of these items raised the KMO from 0.7984 to 0.8368. Data were subjected to principal components analysis using SPSS/PC+ Version 5.0 with results checked against routines in SYSTAT and Stata. Principal components were varimax rotated using the Jolliffe criterion, which is conservative in that more components will be retained than by using alternative criteria, and items will not be prematurely excluded from analysis [5]. All items with component loadings ≥ 0.50 were retained. This led to an additional 3 items or symptoms being dropped from the new tool. The new tool called the Therapy-Related Symptom Checklist (TRSC) has 25 items or symptoms.

The TRSC accounted for 78.8% of the variance in the study sample. Its Cronbach's alpha was 0.85, and it correlated 0.97 with summated symptom concern scores (SC) of patients on the larger 37 item checklist. It discriminated well between patients in radio- and chemotherapy with 79% of patients correctly classified in a linear discriminant analysis. The SC of the TRSC correlated significantly and in the correct direction with the functional status of patients on the Karnofsky scale (r = -0.35, p < 0.001).

To date, all patients and clinicians (physicians and nurses) have reported highly favorable experiences using the TRSC in outpatient clinics.

### Creation of the Therapy-Related Symptom Checklist for Children (TRSC-C)

After the successful use of the TRSC in a number of clinical settings, it was decided to produce a children's version to be called the Therapy-Related Symptom Checklist for Children (TRSC-C) for use in pediatric and adolescent oncology clinics [6]. Funding to produce such a tool was provided by the Alex's Lemonade Stand Foundation in Philadelphia, Pennsylvania, USA. The study to produce a calibrated instrument for children began in 2006. It involved 385 children (5-11 years, n = 222) and teens/adolescents (12-17 years, n = 163) at oncology outpatient clinics in 5 university affiliated children's hospitals in the central, eastern, western, and southeastern USA.

A checklist with 34 symptoms was produced. The same scoring system as with the TRSC was used to score presence and intensity of each symptom. This 34-item list contained most of the 25 items on the TRSC plus other items mentioned in the literature and that the nurses and physicians at the 5 participating institutions believed to be useful for monitoring the symptoms of children with cancer. The items or symptoms printed on the checklist included the name of symptom followed by kid-friendly terms describing the symptom. Data were collected from children and parents participating with their children at the outpatient clinics. Teenagers generally preferred to answer the checklist themselves.

The checklists collected from children and teens were analyzed as follows. After a Bartlett test of sphericity supported the application of factor or principal component analysis to the data, the Kaiser-Myer- Olkin (KMO) was calculated. None of the items or symptoms had a KMO < 0.80; therefore, a principal components factor analysis (pcfa using Stata version 11.1) was done using all 34 items. Factors (components) were retained if they had eigenvalues of 1.00 or greater. After the varimax rotation, items were considered to load on those factors on which their loadings were ≥ 0.40. All but 4 of 34 items possessed adequate loadings and were retained on the new checklist. Therefore, the new TRSC-C has 30 symptoms or items. (See Appendix B, additional file 1)

The Cronbach's alpha of the TRSC-C was 0.91. Summated TRSC-C scores correlated significantly with measures of functional status (r = -0.32, p = 0.02). The correlation of the TRSC-C with a well known measure of pediatric quality of life, the PedsQL, was r = -0.68, p < 0.0001 [7]. The TRSC-C accounted for 53% of the variance in the study sample, since children and teens tended to be somewhat heterogeneous groups. Older patients reported somewhat higher mean symptom concerns on 11 of the 30 symptoms on the checklist. For this reason, it has been suggested that checklist use be examined carefully when answered by children and teens.

## Results

### Use of checklists in different settings

Both checklists have been used in different clinical settings with favorable comments received from clinicians and patients. The TRSC and TRSC-C are available in Spanish language versions, Chinese versions, Pilipino (Tagalog), Bahasa Indonesia, Thai, and are being developed and examined for use in other cultural settings in Europe and Asia. One study is underway in Africa. Clinicians have found that the checklists can be used for anticipatory guidance with patients; that is, discussions with patients can become more focused and deal explicitly with symptom management and treatment concerns.

Recently, a published sequential cohort study done at a Mayo Clinic community based outpatient cancer center has shown that use of the TRSC during treatment can improve the number of symptoms identified and managed in the medical record by 116%, significantly improve (both clinically and statistically) the health related quality of life of patients (HRQL), and significantly improve the functional status of patients [8]. Data also indicate that the number of symptoms documented and managed initially are higher in the treatment group, but decrease over time in the treatment group but not in the control group. This result is consistent with expectations about the use of the TRSC to properly identify and address all important symptoms of concern to patients.

The findings associated with TRSC use are consistent with a call by the World Health Organization (WHO) and others to use checklists to avoid surgical and other medical errors [9]. This call should be extended to include all kinds of services (not only surgery) that can be improved through presentations of simple lists of items or symptoms, procedures, or activities that might enhance patient recall, improve clinician-patient communications, and promote anticipatory guidance collaboratively among patients and clinicians.

Although the TRSC was originally developed to meet needs for better symptom identification and improved clinician-patient communication, the authors and users of the TRSC and TRSC-C have noted that other possibilities for use of the instruments exist. First, the checklists themselves correlate highly with quality of life measures, which suggest that the TRSC might be able to be used as a proxy measure thereby reducing paperwork burdens. Second, although it cannot be discussed in this paper, the TRSC and TRSC-C appear successful in capturing symptom clusters, which is a new and important area in the management and treatment of cancer. Third, the checklists allow symptoms to be systematically monitored across time.

### Conclusions from clinical outcomes reports

Early studies were done of TRSC use at distant clinics using two-way video communications and the collection and storing of data in a computer [10]. These studies indicated that both clinicians and patients were very much in favor of the use of TRSC, since the new checklist was much more clinic-friendly and relevant to treatment than previously used tools. For example, some previously used tools were complex with many interpretation difficulties surrounding questions or items provided to patients. These issues required extended time and discussions about the meaning of questions rather than treatment issues or direct concerns of the patients. Completion of these tools frequently required more than 20 minutes, while the TRSC is easy to complete in only 3-5 minutes. The early TRSC studies also found that computerization allowed clinicians and patients to easily and efficiently review symptoms reported during prior encounters and on-going treatments [10].

### Discussion, implications, and follow-up

Below is a discussion of how the TRSC symptoms (or items) are being used to develop and pilot test an electronic system that will allow frequent communications among clinicians and patients using common language. This system combines computer algorithms with telephone-voice communication capabilities. The system encourages frequent contacts between patients and clinicians rather than relying solely upon fixed clinic visits and completion of paper versions of the TRSC prior to these visits.

### Usable Information Technology (IT) applications

Among the earliest users of the TRSC were geographically remote clinics in Kansas in which two-way video communications technologies were used [10,11]. High levels of clinician and patient satisfaction with TRSC use in these settings as well as more traditional ones led to increased discussions about future online and telecommunications applications of the TRSC and its checklisted symptoms. Now that the TRSC has been shown to adequately capture patient symptoms and has been demonstrated to improve patient outcomes, attention is being given to development and testing of an electronic automated system for use by patients and clinicians. Such a system combined with computer algorithms might be used to flag patients and send these flags to clinicians when symptoms or symptom clusters indicate that such contacts might be helpful.

The system now being piloted incorporates capacities of computers for storage, retrieval, and responses using algorithms. It includes a human voice capability (noted below) to respond to patient inquiries or to make calls to patients over the telephone. Such a system can be used to improve communications among physicians, nurses, and patients preceding or following face-to-face clinic consultations.

Many discussions of the use of electronic systems in the literature remain narrow in that their emphasis is on data collection methods that follow a biomedical interview format that is known to reduce satisfaction of both providers and patients [12]. Better health outcomes are achieved when interviews 1) allow the patient to express his or her needs, 2) the doctor explores symptoms, and 3) the patient is informed [13,14]. There is increasing recognition that a computerized interview process can be therapeutic, particularly when it is used to supplement traditional clinical visits and interventions.

An interview, therefore, can be far more than data collection or documentation. It is an opportunity to tell and demonstrate to patients that they are important and that their symptoms and concerns matter, not the least, in the management of disease symptoms and treatment outcomes. Indeed, the visible and appropriate application of new technologies and methods can demonstrate to patients that steps are being taken to incorporate their concerns into treatment. An interview can encourage and invite patients to actively share information, participate in, and collaborate with clinicians in their treatment [13]. There are increasing reports of successful use of computers in clinical settings, particularly those in which sensitive information is solicited from patients [15-18].

In a computerized electronic environment, patients' adherence to treatment and identification of treatment paths or patterns acceptable to them can be facilitated by the sophistication with which computer algorithms are empathic, convey information accurately, and provide patients with timely answers to questions. In recent decades, there have been many attempts to create computers that can show feelings or affect to patients [19]. Studies of computerized therapy demonstrate that patients can feel better after interacting with a computer [20]. These studies confirm that machines, if properly designed, can be perceived by patients as a helping resource.

The remainder of this paper is a somewhat abbreviated discussion of features that are incorporated into the computerized pilot implementation of the TSRC that is underway. This pilot demonstration was designed to show patients' that their treatment symptoms could be more successfully managed through improved symptom identification using an electronic system and increased collaboration with clinicians about how to alleviate their symptoms. Affective behaviors of the patient are addressed. For example, the electronic system being piloted recognizes that acknowledging patients' symptoms is different from showing sympathy [21]. Recognition validates patients' emotions; sympathy is the sharing of feelings. If a patient reports "I feel awful," a possible way of *recognizing *the symptom is to explore it further. In such instances, the computer can ask: "Tell me more why you feel awful." A sympathetic response shares the feeling by saying: "Sorry to hear that." Recognition verifies how and why the patient feels awful while the later shares the feeling. A machine that shows only sympathy is fundamentally flawed as patients know that machines do not feel. Sympathy from a machine is not credible. On the other hand, machines that show that they have understood the symptoms expressed by the patient fulfill the role that patients expect from these machines, i.e. to explore, record, and report what symptoms the patient faces.

#### How to show understanding

When computers interview a patient about their symptoms, the interview provides an avenue to establish a helping relationship and show that patient symptoms have been recognized by the computer. This section describes specific interviewing techniques the computer can use to show understanding of patients' symptoms.

#### Random change

Patients will differ on which symptom management techniques work best for them. To be viewed as credible, the computer must select and modify interactions with patients based upon patient responses over time. The concerns and treatment symptoms of patients will vary over time, particularly as treatment progresses. The patient may become angry, anxious, in denial, vague, tired, and might want to embellish. The computer needs to recognize the situation and take corrective action. Not surprisingly, this may make each computer interview unique. The techniques that will be described should vary to fit patients' moods and expectations, a task that is difficult and may not always be possible. Techniques used by the computer also should vary randomly just to introduce variety, avoid repetition, and minimize perceptions that the computer is insensitive to the patient's mood. Flexibility in how the computer asks questions suggests to the patient that the interview is tailored to their unique needs and reassures patients that although a computer is being used, they are not being treated merely as an object [13].

#### Changing format

Computers can ask about patient symptoms in different ways.

1. It can ask an open ended question such as "What are the symptoms you are experiencing since our last call?" Patients' responses are matched to keywords.

2. It can ask if the patient has a specific symptom, as in "Do you have fever?" To do so for many symptoms would lead to a long interview.

3. In order to avoid many questions, several symptoms may be combined into a group. The computer might ask about a group of symptoms such as: Did you have fever, vomiting or headache? The computer groups the symptoms together based on previous responses so that they share the same response. At least three methods exist for how symptoms could be clustered together. One could base the decision on probability of the remaining symptoms conditioned on symptoms reported at this point in the interview. It may be based on the history of the patient's reported symptoms. It may be based on population analysis of symptoms and the frequency with which symptoms co-occur.

4. It might change the words used in asking the same question. It might ask "Did you vomit," "Have you vomited," or "Did you throw up?" These get to the same underlying symptom but use different words.

By changing the format of asking questions, a computer can better meet the needs of the patient. While change does not show understanding, a failure to change despite patients' utterances will signal to the patient that the computer has not understood him/her. Changing question formats not only reduces monotony but also reassures patients that the interview reflects their needs.

#### Levels of error response

Computers sometimes do not understand patients' responses, especially open ended ones. In response to the question "what symptoms have you experienced since the last call?" the patient may say words that are not registered correctly, or are symptoms that the computer had not anticipated. In such instances, the patient can feel misunderstood and, if this persists, become frustrated.

There are several techniques to reduce misunderstanding and to prevent frustrations. The machine is perceived as flexible by having different ways to solve a problem. A computer must have incorporated into its programmed code some ways to distinguish the causes of error and respond accordingly. An error may occur if the patient has given an unanticipated response, has not said anything, or if there is too much background noise. If nothing has been clearly stated, the computer gives a new example of what is expected and asks the patient if he/she wants to have more time. If there is too much noise in the background, the computer acknowledges the noise and asks if the patient can press keys on the keypad to continue. If the patient says a word that is not among the keywords used by the computer, then the computer needs to acknowledge the problem and ask for another way of saying the same thing. If the word is still not understood, the computer can record the symptom and have a human being review the recording or transfer the call to an operator for help. Either way, responses to each error message must be uniquely different so that the patient does not feel trapped and forced to abandon the interview. These steps call for error processing routines that can discern the cause of the error.

#### Transitional statements

During the interview process, the computer makes clear transitional statements. For example, "I have reviewed the symptoms you had in our last call. Let me check now if there are any new symptoms." These statements prepare the patient for what is coming next, shows progress, and acknowledges what has occurred so far. Transitional statements are common in conversations and are a necessary step for meeting patient's expectations [22].

#### Explicit verification

A computer that is collecting data on patient's symptoms can explicitly show understanding by checking with the patient that the recorded symptom is correct. This can be done by repeating the same words used by the patient or, preferably, use alternative words that have the same meaning. Explicit verification of symptoms is very mechanical and re-wording is often used to break the monotony. For example, when a patient reports "I threw up last night" the computer may validate this feeling by saying "Last night you vomited." In our pilot study, we ask the patient an open ended question: "Tell us how you felt since our last call?" The response to this open-ended question is verified by standardized words used by the machine. If the patient says: "I felt tired." The computer matches the word "tired" and classifies it as "fatigue" and might say: "You are reporting fatigue." Finally, computers can provide a summary of symptoms collected from the patient. A statement such as "So far, you have reported that you have fatigue and vomiting," is also an example of explicit verification.

### Signs of deliberation

The length of the pause gives the impression that the computer is thinking through the patient's statement. The computer might say: "I see." Or, it might say: "I am going to come back to this later." These and other similar statements suggest that an important statement has been made by the patient.

### Connecting to history

In this approach, the patient's symptom is validated by connecting it to the patient's history. The computer might say: "This is a new symptom." Or it might say: "You also mentioned fever last time. It seems you continue to suffer from it." A history shows to the patient that not only the computer has understood the current symptom but it also has captured and understood past reports.

### Asking for more details

Asking for more details shows the patient that they are being heard. When the patient says "I threw up" the computer might respond: "How severe was it?" "Was it after dinner?" or "Was it a projectile?" In our pilot, we routinely asked about the severity of a symptom after each symptom. In addition, computers can ask about chronology, bodily location, quality, quantity, setting, any aggravating or alleviating factors, and associated manifestations. The details may or may not matter but asking about them indicates that the original report of the symptom was understood.

### Suggesting coping methods

One way to acknowledge the presence of a symptom is to suggest methods that can be used to cope with the symptom. For example, if the patient reports vomiting, then the computer can ask "I see that you were prescribed pills for vomiting. These are the small blue pills. Are you taking these pills?" These additional statements reassure the patient that their reported symptom has been understood. If symptoms are considered very severe, or if symptoms are a threat to successful treatment outcomes, the patient can be put in touch with a clinician through telephone and by computer generated messages. Clinicians also can instruct the computer to more frequently and explicitly monitor a particular symptom of concern.

### Active listening

Computers can listen tirelessly to the patient but when the patient pauses it is important to use active prompts to encourage additional comments. After the patient has reported one symptom, the computer can prompt for more detail by re-wording the patient's reported symptom into a question. It may ask "You felt short of breath?" A pause after such a question would solicit more details. Whether these details are on the checklist of symptoms is not material when improved communications is a primary objective. The fact that the patient provides these details confirms to the patient that the computer has understood earlier reports of the symptom. Active listening might also take the form of short phrases such as "Tell me about another symptom?" or "Tell me more" for short.

### Getting to "Yes"

An important way to reassure patients that they have been understood is to anticipate their responses by asking leading questions. Clinicians often do this. Early in clinic visits clinicians ask general questions but later during the same visit good clinicians ask questions that anticipate patients' responses [23,24]. Leading questions signal that the clinician has arrived at conclusions. A computer can do the same. It can change questions into declarative statements and follow these with brief verifications. For example, if the computer, based on the patient's history, anticipates that the patient has fever, it may change from "Do you have fever?" to "You still have fever, right?" The response to these leading questions is organized to be "Yes." It signals to patients that the computer is aware of their condition.

### Reporting on human connection

An easy way for computers to tell the patient that they are being understood is to report that their data has been communicated to their clinicians. Even if the data are not yet examined by a clinician, the computer needs to inform the patient. A statement such as "The symptoms you reported in the last call were sent to the office of X" reassures the patient. When the clinician indicates need for closer monitoring of a symptom, the computer can also alert the patient with a statement such as: "Your doctor [or nurse] was concerned about your fever and wanted us to call this morning to verify that it has subsided."

### A few technical details concerning the pilot study

The TRSC computer interview works on an Interactive Voice Response telephone system. It has extensive orientation statements, alerting the patient of passage from one section of the interview to another. It reviews symptoms reported in past calls one-by-one and provides a summary of its findings at end of the section. It then groups remaining symptoms into cohesive categories or clusters and asks about both individual symptoms and clusters. It ends with an open question: What other symptoms have you experienced since our last call?" It matches responses to keywords and acknowledges the responses selectively. The system is built on progressive error messages that transfer the call to human operator if the system fails to address the error. It uses active listening techniques to solicit more symptoms after open ended questions. It reports to the patient the extent to which symptoms have been reported to his/her clinicians. It asks leading questions towards the end of the interview. It changes the format of asking questions and the words used to describe a symptom. It explores details of symptoms asking for severity or other features of the symptom. The TRSC computer interview is now at early phases of creating a computerized interview that mimics real conversation with undertones and meaning not only in what is said but what is not said as well.

## Conclusions

Data on the impact of these innovations on patients' health care outcomes will be collected in a larger study following completion of the pilot now in progress. A comprehensive but useable theory concerning what makes a computer interview more empathetic and clinically relevant has not been developed. Such a theory may emerge from this pilot and additional work with the system now being developed. The system now incorporates many suggestions from the literature, but it and study findings should be of increasing interest to clinicians and information scientists as more experience with its use is obtained.

## List of abbreviations used

**HRQL **Health Related Quality of Life

**KMO **Kaiser-Myer-Olkin

**PedsQL **Pediatric Quality of Life Inventory

**SC **Symptom Concern Score, summed score from the TRSC or TRSC-C

**TRSC **Therapy-Related Symptom Checklist for Adults

**TRSC-C **Therapy-Related Symptom Checklist for Children

## Competing interests

The authors declare that they have no competing interests.

## Authors' contributions

All authors were involved in the drafting and revising of the manuscript for critical intellectual content. All have given final approval for the version to be published and agreed to be accountable for all aspects of the study and manuscript.

Conception and design of the manuscript are the primary responsibility of ARW, PDW, FA, but all authors have contributed.

Data analysis was contributed by DDW, ARW, FA.

The primary development of computer algorithms and voice systems were BD, FA.

Evaluations of computer algorithms for the pilot study were done by BD, FA, HH, REK, PDW, ARW.

## Declaration

Publication costs for this article were funded through a research incentive account of Dr. AR Williams at the University of South Florida, Tampa, Florida.

This article has been published as part of *BioMedical Engineering OnLine *Volume 14 Supplement 2, 2015: Select articles from the 2nd International Work-Conference on Bioinformatics and Biomedical Engineering (IWBBIO 2014). The full contents of the supplement are available online at http://www.biomedical-engineering-online.com/supplements/14/S2.
